# P268S in *NOD2* associates with susceptibility to Parkinson’s disease in Chinese population

**DOI:** 10.1186/1744-9081-9-19

**Published:** 2013-05-07

**Authors:** Qilin Ma, Xingkai An, Zhiming Li, Huanjing Zhang, Wenqing Huang, Liangliang Cai, Peng Hu, Qing Lin, Chi-Meng Tzeng

**Affiliations:** 1 Department of Neurology, The First Affiliated Hospital of Xiamen University, Xiamen, Fujian 361003, China; 2 School of Pharmaceutical Sciences, Xiamen University, Xiamen, Fujian 361005, China

**Keywords:** Parkinson’s disease, Variants, P268S

## Abstract

**Background:**

The cause of almost all cases of Parkinson’s disease (PD) remains unknown. Recent years have seen an explosion in the rate of discovery of genetic defects linked to PD. Different racial and geographical populations may have different distributions of genetic variants.

**Methods:**

In the current study, we screened the following genetic variants, including some rare mutations and single nucleotide polymorphisms (SNPs), in a pedigree and cases-controls. To best of our knowledge, we first screened these variants known to be associated with neurodegeneration disease, E46K (rs104893875) in *SNCA*, A1442P in *LRRK2*, IVS9 in *PARK2*, A350V in *SLC41A1*, P268S (rs2066842), R702W (rs2066844), G908R (rs2066845), 1007fs (rs2066847) in *NOD2* and G2385R (rs34778348) in *LRRK2* from southern China population. Genotyping was performed by jointly using primers overlapping polymerase chain reaction (PCR) site-directed mutagenesis, restriction fragment length polymorphism (RFLP), and capillary electrophoresis (CE).

**Results:**

We didn’t discover above 9 variants in the family members of the pedigree. Furthermore, of 237 patients with sporadic Parkinson’s disease and 190 controls, no heterozygosity or homozygosity were found from E46K, A1442P, A350V, R702W, G908R, or 1007fs but heterozygosity onto G2385R, IVS9, and P268S. No significant difference between cases and controls was found in both allele frequency (*P* = 0.572) and genotype frequency (*P* = 0.348) of IVS9. However, significant differences in genotype frequency (*P* = 0.009) of G2385R were consistent with prior observation. Eight patients with Parkinson’s disease (2 women and 6 men are over the age of 50 years at onset of PD) carried the P268S heterozygous variation in *NOD2*. There was no heterozygosity or homozygosity of P268S in the controls. Genotype frequency of P268S (*P* = 0.0450) had significant differences.

**Conclusions:**

Our results suggested that the P268S variant in *NOD2* might be a risk factor for susceptibility to sporadic Parkinson’s disease in Chinese populations. It also implied that the inflammatory response may play a role in PD.

## Background

Parkinson’s disease (PD) is a complex neurodegenerative disease caused by a variety of factors. The incidence in the population over the age of 65 is about 1.8% [1]. The major clinical features of PD include resting tremor, bradykinesia and rigidity [2]. The pathological features of PD include progressive loss of dopaminergic neurons from the substantia nigra pars compacta and the presence of intercellular Lewy bodies in surviving neurons [3]. The cause of PD is unclear, but it is generally considered to be associated with the impairment in degradation of ubiquitin proteasome system (UPS), mitochondrial dysfunction, and oxidative stress [4-6]. We hypothesize that genetic and environmental factors and aging together lead to the development of PD. In this study, we investigated five genes, including *SNCA*, *LRRK2*, *PARK2*, *SLC41A1*, and *NOD2*, all of which might be involved in the cytological mechanisms of neurodegeneration disease aiming to identify genetic variants associated with sporadic PD in Chinese patients and understand the genetic etiology of PD.

The first gene to be identified as associated with PD was *SNCA* (*PARK1/4*), which encodes α-synuclein. The major mutations in *SNCA* include A53T, A30P, and E46K [7-9]. Although these mutations account for less than 1% of the cases, patients carrying these mutations have obviously clinical phenotypes [8]. The A53T and A30P mutations have been found in Chinese patients with sporadic PD [10]. However, the association of E46K with sporadic PD has not been reported in Chinese patients.

*LRRK2* (*PARK8*) encodes a large protein containing five functional domains, involved in a number of physiological functions, including substrate binding, protein phosphorylation and protein interactions [11]. The most common mutation G2019 of *LRRK2* in European populations presents less than 0.1% in Asian individuals [12]. The G2385R was at a significantly higher frequency in Asian patients than in controls [13]. One of the purposes of this study is to re-validate this variant in southern China. In addition, Yue Huang found A1442P in pedigrees of the Australian patients and considered it pathogenic, because it is conserved across many species and a substitution of Ala with Pro can change the secondary structure of proteins [14]. However, A1442P has not been further confirmed from a large number of cases and other populations.

The most common cause of young-onset sporadic PD is autosomal recessive *PARK2* mutation [15]. The E3 ubiquitin ligase, parkin, which is encoded by *PARK2*, can specifically degrade UPS through tagging ubiquitin on protein [16]. Exon deletion, insertion, and point mutations in *PARK2* have been found in different ethnic groups [17]. In 2009, Yih-Ru Wu screened 506 Taiwan sporadic patients with age of onset below 50 years for *PARK2* gene mutation and identified a novel IVS9 insertion (c.1084intron^+^) [18]. The c.1084intron^+^ was due to a G > A polymorphism at position −6 of a cryptic splice acceptor site within IVS9.

In recent years, genome-wide association studies (GWAS) have identified a number of new susceptibility loci associated with PD in different ethnic groups. Of these loci, the most striking locus is *PARK16*, which is located in 1q23 and contains 5 genes (*SLC45A3*, *NUCKS1*, *RAB7L1*, *SLC41A1*, and *PM20D1*) [19]. *SLC41A1* encodes a 56 kDa Mg^2+^ transporter consisting of 513 amino acids, it has been proposed that PD might be associated with lack of Ca^2+^ and Mg^2+^ in the brain [20]. In 2010, Arianna Tucci sequenced *PARK16* in 182 patients with PD in the United Kingdom and found A350V in *SLC41A1*, but failed to detect it in a large series of ethnicity-matched controls (n = 483) [19]. To inspect whether this coding mutations may or may not be the disease-causing mutation, we aim to screen A350V in Chinese Han populations.

Several obtained findings suggest that inflammation also contributes to the pathogenesis of PD [21-23]. It has been reported that the G174C variant in the *IL-6* promoter may influence the risk for developing PD, particularly regarding early age of onset PD [24]. Recent studies revealed that NF-κB-mediated inflammation might also play an important role in the pathogenesis of PD [25,26]. NOD (nucleotide-binding oligomerization domain) proteins, i.e. NOD1 (a product of *CARD4* gene) and NOD2 (encoded by *NOD2*), are intracellular signaling molecules that recognize bacterial components, mediate the activation of NF-κB and induce or enhance apoptosis [27]. In 2007, Monika Bialecka et al. screened 308 Portland patients for 3 variants (R702W, G908R, and 1007fs) in *NOD2* which were associated with Crohn’s disease (CD) in Europeans. They found that *NOD2* might be associated with susceptibility to PD [28]. Although inflammatory response has long been considered as one of the factors for PD development, it has not yet been reported that R702W, G908R, 1007fs and P268S in *NOD2* is associated with Chinese PD patients.

In this study, we screened 237 patients with sporadic PD and 190 controls for the 9 variants, E46K (rs104893875), G2385R (rs34778348), A1442P, IVS9, A350V, R702W (rs2066844), G908R (rs2066845), 1007fs (rs2066847) and P268S (rs2066842), in 5 genes which are possibly found to be associated with Chinese PD patients, including *SNCA*, *LRRK2*, *PARK2*, *SLC41A1*, and *NOD2*. These variants included both rare mutations (e.g. E46K and A1442P) and SNPs, so we also screened them in a pedigree with two PD cases. In this way, we aimed to provide references for the study of disease-causing or susceptibility gene for PD. In addition, using the overlap extension polymerase chain reaction (PCR)-based site-directed mutagenesis, we constructed wild-type and homozygous mutant plasmids for these variants, which were used as controls to determine variants in this study.

## Material and methods

### Study subjects

A total of 237 sporadic patients with PD (94 women and 143 men) and a pedigree with two PD cases were recruited from Xiamen First Hospital, Fujian Province, China. The average age of enrollment was 60.3 ± 11.3, ranging from 25 to 83 years. Their average age of onset was 56.4 ± 10.8 years, ranging from 23 to 80 years. Of these patients, 51 (21.5%) had early-onset PD (EOPD), defined as the age at onset <50 years; 186 (78.5%) cases had late-onset PD (LOPD), with age of onset ≥ 50 years. Patients were independently diagnosed by two neurologists. Diagnostic criteria were taken from the United Kingdom PD Brain Bank [29]. The severity of the disease was determined using the Unified Parkinson’s Disease Rating Scale (UPDRS). The control group consisted of 190 individuals (75 women and 115 men), with an average age of 51.7 ± 9.8 years (ranging from 23 to 80 years). The cases and controls were matched with respect to age, gender, and place of residence. This study was approved by the Xiamen First Hospital Ethics Committee. Informed consent was obtained from all participants.

### Genomic DNA extraction

Five milliliters of peripheral blood with ethylene diamine tetraacetic acid (EDTA) anticoagulant was collected from each participant. Genomic DNA was extracted using the MagCore Genomic DNA Whole Blood Kit and HF-16 extractor (Cat. No. MGB400-04, RBC Bioscience Taiwan) according to the manufacturer’s instructions and stored all samples at −20°C before use.

### PCR amplification

Primers (Table 1) were designed using the software Primer Premier 5 according to the change of restriction sites, caused by the mutation. Primers were synthesized by Sangon Biotech (Shanghai, China).

**Table 1:** Primer sequences, PCR conditions and restriction digestion predictions

**Polymorphism**	**Primer sequences (5′ → 3′)**	**Annealing temperature (°C)**	**Restriction enzyme**	**Amplified products length (bp)**	**Restriction products length (bp)**
E46K	F: TGATGTGGGAACAAAGGGGA	58	*BsaJ*I	747	46E allele: 287,460
	R: GTGTTTCCTGAAATGCACTCTGA				46K allele: 747
A1442P	F: GAGACTAAACTGCTGCTTGC	58	*Hha*l	801	1442A allele: 671,130
	R: GTAATCTCGTATGGCAGGGA				1442P allele: 801
A350V	F: TCAGTGGTCTTTGCGTCATT	58	*Mwo*I	302	350A allele: 94,208
	R: CTGTCCTTTTACTCTGCTCCC				350V allele: 302
P268S	F: AGCCCATTGTCTGGTTAGGT	58	*BamH*I	309	268P allele: 309
	R: ACAGTGTCCGCATCGTCAT				268S allele: 225,84
R702W	F: AGATCACAGCAGCCTTCC	63	*MspI*	185	702R allele: 20, 35, 54, 76
	R: CACGCTCTTGGCCTCACC				702W allele: 20, 35, 130
G908R	F: CCCAGCTCCTCCCTCTTC	63	*Hin6I*	380	908G allele: 380
	R: AAGTCTGTAATGTAAAGCCAC				908R allele: 242, 138
1007fs	F: GGCAGAAGCCCTCCTGCAGGGCC	58	*ApaI*	151	Wild type allele: 151
	R: CCTCAAAATTCTGCCATTCC				1007f allele: 130, 21
IVS9*	F: ACTCCTGCGCTTGATTTAGGCAAT	58	*Xho*l	775	Wild type allele: 318,457
	R: TTGGAATTTAGCTGTTCCTTCGGG				IVS9 G → A allele: 775
G2385R	F: AGACACTGCTCTCTATATTGCTAAG	58	*Acc*I	261	2385G:261
	R: CTGAAAAGATGGTGCTGAGAAG				2385R:77,184

*: IVS9: Intervening sequence 9, *PARK2.*

### Restriction endonuclease analysis

Target variants were detected using the PCR-RFLP (restriction fragment length polymorphism) method. The digestion reaction and restriction enzyme digestion conditions were taken from the manuals included with the restriction enzymes purchased from the New England BioLab (United States). Then 20 μl of digestion products were loaded on 2% agarose gel containing ethidium bromide, electrophoresed under 100 V for 30 min in 1× TAE, and imaged using the gel imager. Digestion results of homozygous mutants, heterozygous mutants, and wild-type are shown in Table 1.

### DNA sequencing

Based on the results from PCR-RFLP and site-directed mutagenesis, the target fragments were re-amplified using PCR. PCR products were purified using a DNA gel purification kit, sequenced using the dideoxy four-color fluorescence method on an automatic sequencer (ABI, U.S.), and compared using GenBank data to confirm the mutation sites.

### Capillary electrophoresis (CE)

In this study, we initially established the test method using CE to detect gene polymorphisms for PD. Briefly, based on the susceptibility gene polymorphisms of PD identified from PCR-RFLP, restriction fragments were detected using capillary electrophoresis (BIOptics Qsep100 dna-CE, Taiwan), which used a molten molding silica capillary column with an inner diameter of 75 μl and column length of 150 mm and Mops-Tris buffer of pH 7.55. Then 0.5 μl of sample was electrically injected in 4kV × 15 s at 25°C, electrophoresed at a constant voltage of 8 Kv, and detected for the laser-induced ethidium bromide emission wavelength at 590 nm. The gel electrophoresis profiles were compared to determine the genotypes.

### Statistical analysis

SPSS 18.0 (Statistical Package for the Social Sciences, version 18.0 for Windows) was used for statistical analysis. Allele and genotype frequencies were compared between cases and controls using the Fisher’s exact test. To avoid multiple easily false, false discovery rate was used to correct p values. Odds ratios and 95% confidence intervals were calculated. A two-sided *P* value ≤0.05 was considered statistically significant. Power was calculated by Power and Sample Size Calculations Version 3.0, 2009.

## Results

### Pedigree

**Figure 1:**
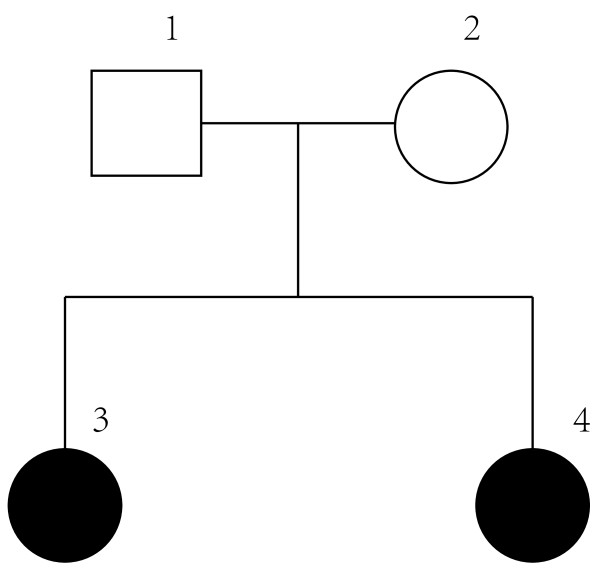
**Pedigree of Parkinson’s disease (PD).** Circle, woman; square, man; filled square, affected.

Figure 1 shows the pedigree (2 generations) of the family that we studied. The age of PD onset for the affected individuals 3 and 4 in the kindred is 45 and 40, respectively. We failed to discover any mutations of the 9 genetic variants, including rare mutations E46K and A1442P originally found in the kindred. It may be caused by low family members to detect the association. Further studies using a larger Chinese pedigree with PD patients are required to determine this.

### Case–control study

**Figure 2:**
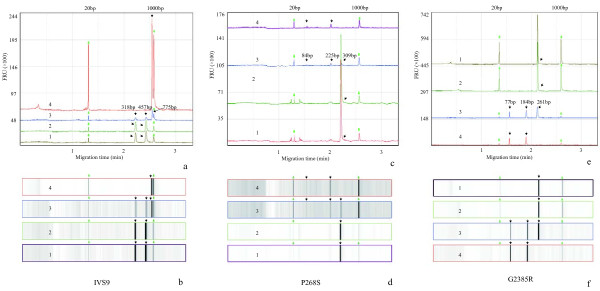
**Capillary electrophoresis (CE) results of restriction fragments of 3 variants. a**, **c**, **e** and **b**, **d**, **f** are the electropherograms and gel-views of IVS9, P268S, and G2385R after restriction digestion, respectively. The green arrows indicate markers (20 bp, 1000 bp) and black arrows indicate the restriction fragments. Restriction fragment sizes are shown in the Table 1: No mutation (wild-type/negative control), 2: Negative results in the control group, 3: Positive results (heterozygote) in the case group, 4: Homozygous mutation (homozygote/positive control). Because the signals are too strong for IVS9-4, P268S-1, P268S-2, G2385R-1, and G2385-2, 10 times markers are used to enhance the signal.

We analyzed 9 variants, E46K, G2385R, A1442P, IVS9, A350V, P268S, R702W, G908R, and 1007fs in 237 sporadic patients with PD and 190 controls using the PCR-RFLP and CE. All the genotypic and allelic distributions analyzed in this study were in accordance with the Hardy–Weinberg equilibrium. No heterozygosity or homozygosity of E46K, A1442P, A350V, R702W, G908R, or 1007fs was found. Heterozygosity of G2385R, IVS9, and P268S were found in the case groups and controls (Figure 2). The genotype frequency, allele frequency, odds ratios, and confidence interval of these variants are shown in Table 2.

**Table 2:** Genotype and allele frequencies of G2385R, P268S, and IVS9 in cases and controls

**Genotype**	**PD patients, n = 237**		**Control, n = 190**		Odds ratio (95% CI)*	p value**	**Power**^**§**^
	**No.**	**%**	**No.**	**%**			
G2385R(G > A)							
GG	213	89.9	186	97.9	1		
GA	24	10.1	4	2.1	5.24	0.009	0.8973
					(1.79-15.38)		
AA	0	0	0	0	—	—	
G	450	94.9	376	98.9	1		
A	24	5.1	4	1.1	5.01	0.090	0.8803
					(1.72-14.58)		
P268S(C > T)							
CC	229	96.6	190	100	1		
CT	8	3.4	0	0	—	0.045	NA
TT	0	0	0	0	—	—	
C	466	98.3	380	100	1		
T	8	1.7	0	0	—	—	
IVS9(G > A)							
GG	207	87.3	175	92.1	1		
GA	30	12.7	15	7.9	1.7	0.348	0.2987
					(0.88-3.24)		
AA	0	0	0	0	—	—	
G	444		365	96.1	1		
A	30	6.3	15	3.9	1.64	0.572	0.2865
					(0.87-3.10)		

*: Odds ratios (OR) and 95% confidence intervals (95% CI) were calculated by comparison to the common allele frequencies and genotype frequencies.

**: Fisher exact test and false discovery rate were used to calculate and correct the p values, respectively. Two sided *P* value ≤ 0.05 was considered statistically significant.

^§^: Power was calculated by Power and Sample Size Calculations Version 3.0, 2009. NA: not available.

The distribution of G2385R heterozygosity in *LRRK2* was significantly different between cases and controls (10.1% *vs.* 2.1%, *P* = 0.009). The intron IVS9 heterozygous variant in *PARK2* was found in both the case group (12.7%) and the control group (7.9%). Although this variant had a higher frequency in the case group than the control group, the difference was not statistically significant (*P* = 0.348).

Although only 8 out of 237 patients were found to carry the P268S heterozygous variant in *NOD2*, all these patients (2 women and 6 men) had age at onset of over 50 years. Since no controls were found to carry this variant, the difference between cases and controls was slightly significant (*P* = 0.0450). These results suggest that the variant P268S in *NOD2* might be associated with late-onset PD and is one of the risk factors for sporadic PD in Chinese Han populations.

## Discussion

Gene polymorphism is a risk factor of PD, and it shows different distributions in different ethnic groups and geographies. Although GWAS have identified a number of susceptibility genes and relevant variants associated with PD in different populations, few of them have been analyzed in other populations. To best of out knowledge, we first screened 8 variants that have been associated with neurodegeneration, E46K, A1442P, A350V, IVS9, P268S, R702W, G908R, and 1007fs in Chinese populations, and further verified the presence of the high-frequency variant G2385R in southern China.

In case–control study, both PD patients and controls carried the heterozygous G2385R mutation, and the genotype difference was slightly significant. This is consistent with the results of a previous study of 600 Chinese PD patients and 334 controls [30]. This variant was initially reported by Xingkai An. in 2008 among PD patients in Sichuan area [30]. However, we did not find E46K, A1442P, or A350V variants in patients with PD and in controls. All of them indicated following phenomenon: the ancestry effect; familial PD results from rare, highly penetrant pathogenic mutation (e.g. E46K, A1442P); multiple variants of low penetrant (e.g. A350V) contribute to the risk of PD and are involved in the etiology of PD.

Interestingly, none of homozygosity of these variants was found in either the case or control groups [31,32]. It was reported that heterozygosity of autosomal recessive genes was very important in the initiation and development of PD. For example, it has been found that patients with PD who carry heterozygous *PARK2* and *PINK1* mutations have an age of onset between that of wild-type individuals and patients with homozygous mutations [33,34]. Heterozygous mutation carriers indeed show preclinical changes at the metabolic, structural, or functional levels that are detectable by modern techniques of neuroimaging and electrophysiology. Heterozygosity for putative recessive mutations could lead to disease expression by at least three mechanisms which are haploinsuffciency, dominant-negative effect and novel gain-of-function [35]. Taken together, heterozygous mutations in putative recessive genes seem to increase the susceptibility to develop PD.

The new insertion mutation, c.1084intron^+^ in *PARK2*, introduces two new amino acids and a TAA stop codon, resulting in reduced levels of parkin protein [36]. This insertion mutation may be associated with the -6G > A polymorphism of intron 9 in *PARK2*. One case–control study found IVS9G > A to be a risk factor of PD in Taiwan. A functional study of IVS9G > A by Guey-Jen Lee-Chen showed that the shear efficiency of -6A was slower than that of -6G at the protein level [37]. 12.7% PD patients and 7.9% controls carried the heterozygous IVS9 mutation, but the allele difference and genotype difference were not statistically significant. This result is consistent with the results of a previous study of 506 patients and 508 controls in Taiwan [18]. Further investigation into the association between IVS9G > A and PD need, through larger sample size from various populations.

Inflammation is one of the suspected theories of PD. The activation of NF-κB and microglial cells around dopaminergic neurons have been analyzed in patients with PD [38]. In 2007, Monika Bialecka reported that three variants (R702W, G908R, and L1007fsinsC) of *NOD2* encoding NOD2, a protein that can activate the apoptosis response, were significantly associated with PD in a Polish population [28]. It has been demonstrated that these three variants are not associated with CD in Chinese Han, Korean, and Japanese populations [39-42]. Similarly, these three common SNPs (R702W, G908R, and 1007fs) were not detected in our cohort. However, a novel variant P268S was observed, which has also been discovered to be associated with CD in Chinese population [43]. Although linkage disequilibrium was observed between P268S and the other three SNPs in *NOD2*, only P268S of the four variants was slightly significant in our study, which was consistent with recent study suggesting that P268S instead of 1007fs, G908R, R702W was a risk factor of Chinese Crohn’s disease population. It indicated that P268S may be one of risk factors for PD in Chinese individuals, or the P268S variant may be in linkage disequilibrium with another causal rare variant on *NOD2* gene. The frequencies of minor allele 1007fs, G908R, R702W and P268S variants are much lower in Asians compare to Caucasians populations. In this study, genotype frequency of P268S (*P* = 0.0450) had slightly significant differences. The sample size has to be very large or odds ratio has to be very high to have enough power for detection effect of very rare variants.

NOD2 is a member of the Apaf-1 superfamily of apoptosis regulators that is expressed in monocytes and involved in the activation of NF-κB, by bacterial component muramyl dipeptide (MDP) [44]. Functional studies revealed that both normal and P268S NOD2 induced similar levels of NF-κB activation in response to MDP [45] and the G908R, R702W, and L1007fsinsC variants in the presence and absence of P268S are defective in their ability to respond to bacterial lipopolysaccharides and peptidoglycan, whereas P268S alone exhibited wild-type activity [46]. Thus, P268S was confirmed as the haplotype background of these three variants but has no influence on functions of the CD-associated variants of *NOD2*[47]. Notably, above results from NF-κB activation assay using human embryonic kidney (HEK) 293 cell lines, but the function activity of NOD2 in neurodegeneration field remain unclear. Additionally, Crane et al. found that carriage of the P268S variant was associated with greater disease activity and inversely associated with ulcerative colitis spondylarthritis [48]. We regarded the P268S of *NOD2* behaving as a common factor of PD and CD. Crohn’s disease is a chronic inflammatory disorder of the gastrointestinal tract, which is thought to result from the effect of environmental factors in a genetically predisposed host [49]. A wealth of new information has emerged to suggest that inflammation-derived oxidative stress and cytokine dependent toxicity may contribute to nigrostriatal pathway degeneration and hasten progression of disease in humans with idiopathic PD. Both them suggest that inflammation may play a role connecting between PD and CD.

## Conclusions

We analyzed the genetic loci associated with PD in the sporadic and family PD patients using PCR-RFLP and found P268S might be a risk factor of sporadic PD in the Chinese population. As it is the first study on *NOD2* P268S in PD patients, the results should be treated with caution, as one cannot completely exclude that they are false positive. Further studies required to verify the association between P268S and susceptibility to sporadic Parkinson’s disease in large sample size, and explore the NOD2 functions involved in neurodegenerative diseases.

## Abbreviations

PD: Parkinson’s disease; SNPs: Single nucleotide polymorphisms; UPS: Ubiquitin proteasome system; PCR: Polymerase chain reaction; RFLP: Restriction fragment length polymorphism; CE: Capillary electrophoresis; GWAS: Genome-wide association studies; CD: Crohn’s disease; NOD: Nucleotide-binding and oligomerization domain; EOPD: Early-onset PD; LOPD: Late-onset PD; UPDRS: Unified parkinson’s disease rating scale; EDTA: Ethylene diamine tetraacetic acid; SPSS: Statistical package for the social sciences; OR: Odds ratios; CI: Confidence intervals; MDP: Muramyl dipeptide; NFAT: Nuclear factor of activated T-cells.

## Competing interests

The authors declare that they have no competing interests.

## Authors’ contributions

QLM, XKA, and QL collected the blood samples from PD patients and controls, extracted genomic DNA, and searched relevant literature. ZML performed PCR, RFLP, and CE analysis and built positive and negative control plasmids. HJZ and LLC designed the primers for PCR, cloning, and site-directed mutagenesis and performed sequence alignment. WQH and PH participated in statistical analysis and the production of tables. CMT designed all the experiments and drafted the manuscript. All authors read and approved the final manuscript.

## Authors’ information

QLM: MD, chief physician of the Department of Neurology of the First Affiliated Hospital of Xiamen University; senior visiting scholar at Harvard Medical School, Center of Neurological Diseases; Member of the World Stroke Organization (WSO). XKA: Resident of the Department of Neurology of Xiamen First Hospital; part of the team that first revealed the association of G2385R and the *LRRK2* gene with PD in Chinese Han populations. QL: MD, associate-chief physician of the Department of Neurology of the he First Affiliated Hospital of Xiamen University. ZML: Master student, School of Pharmaceutical Sciences Xiamen University; participated in several projects on screening the disease genes for PD of Chinese Han population. CMT: PhD in Physical Biochemistry, Nuclear Science, National Tsing-Hua University, Postdoctoral Fellow in Biochemistry, Stanford University School of Medicine, School of Pharmaceutical Sciences; Executive Director Professor of Center for Translational Medicine, Xiamen University.
